# Ethyl­enediammonium dichloride

**DOI:** 10.1107/S1600536809018327

**Published:** 2009-05-20

**Authors:** Milad Gabro, Roger A. Lalancette, Ivan Bernal

**Affiliations:** a Carl A. Olson Memorial Laboratories, Department of Chemistry, Rutgers University, Newark, NJ 07102, USA

## Abstract

The title ionic compound, C_2_H_10_N_2_^2+^·2Cl^−^, crystallizes with a center of symmetry within the cation. Each of the positively charged ammonium ends of the mol­ecule is trigonally hydrogen bonded to three different chloride counter-ions, while each of the chloride ions is trigonally hydrogen bonded to three different ethyl­enediammonium cations. The hydrogen-bonding network leads to stabilization of the structure.

## Related literature

For the applications of ethyl­enediamine, see: Kotti *et al.* (2006 ▶); Warner (1912 ▶).
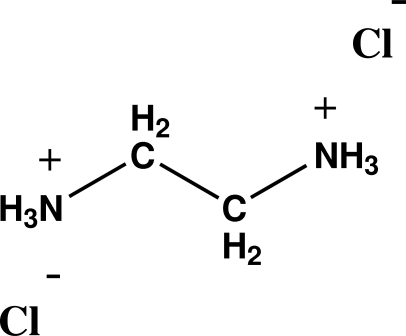


## Experimental

### 

#### Crystal data

C_2_H_10_N_2_^2+^·2Cl^−^
*M* *_r_* = 133.02
Monoclinic, 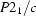

*a* = 4.3807 (3) Å
*b* = 6.8569 (4) Å
*c* = 9.9464 (5) Å
β = 91.192 (2)°
*V* = 298.71 (3) Å^3^
*Z* = 2
Cu *K*α radiation
μ = 8.71 mm^−1^
*T* = 100 K
0.45 × 0.30 × 0.29 mm

#### Data collection

Bruker SMART CCD APEXII area-detector diffractometer
Absorption correction: multi-scan (*SADABS*; Sheldrick, 2001 ▶) *T*_min_ = 0.085, *T*_max_ = 0.090
1654 measured reflections
521 independent reflections
520 reflections with *I* > 2σ(*I*)
*R*_int_ = 0.022

#### Refinement

*R*[*F* ^2^ > 2σ(*F* ^2^)] = 0.028
*wR*(*F* ^2^) = 0.070
*S* = 1.15
521 reflections
44 parameters
Only H-atom coordinates refined
Δρ_max_ = 0.41 e Å^−3^
Δρ_min_ = −0.31 e Å^−3^

### 

Data collection: *APEX2* (Bruker, 2006 ▶); cell refinement: *APEX2*; data reduction: *SAINT* (Bruker, 2005 ▶); program(s) used to solve structure: *SHELXTL* (Sheldrick, 2008 ▶); program(s) used to refine structure: *SHELXTL*; molecular graphics: *SHELXTL*; software used to prepare material for publication: *SHELXTL*.

## Supplementary Material

Crystal structure: contains datablocks I, global. DOI: 10.1107/S1600536809018327/lh2823sup1.cif

Structure factors: contains datablocks I. DOI: 10.1107/S1600536809018327/lh2823Isup2.hkl

Additional supplementary materials:  crystallographic information; 3D view; checkCIF report

## Figures and Tables

**Table 1:** Hydrogen-bond geometry (Å, °)

*D*—H⋯*A*	*D*—H	H⋯*A*	*D*⋯*A*	*D*—H⋯*A*
N1—H1*E*⋯Cl1	0.89 (2)	2.27 (2)	3.1514 (15)	175 (2)
N1—H1*D*⋯Cl1^i^	0.80 (3)	2.39 (3)	3.1770 (15)	170 (2)
N1—H1*C*⋯Cl1^ii^	0.91 (2)	2.29 (2)	3.1922 (15)	171 (2)

Symmetry codes: (i) 
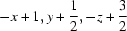
; (ii) 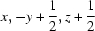.

**Fig. 1.**
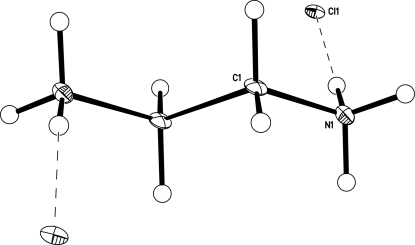
The molecluar structure of (I), with its numbering; one-half of the molecule is generated through a center of symmetry at 1/2,1/2,1/2 in the chosen unit cell. The H bonds to the chlorides are shown as dashed lines. Displacement ellipsoids are drawn at the 80% probability level.

**Fig. 2.**
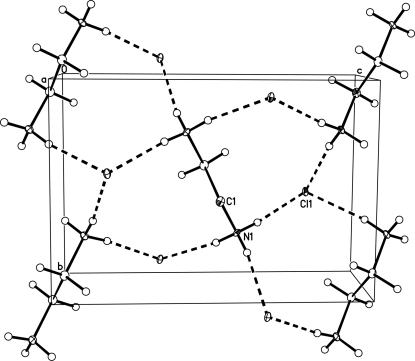
A partial packing diagram for (I), with extracellular molecules, illustrating the trigonal hydrogen bonding of the chloride ion to three different ethylenediammonium cations. Also the three H atoms of the ammonium are bound to three different chloride counterions. All of the cations lie on centers of symmetry, at 1/2,1/2,1/2 and 1/2,0,0. Displacement ellipsoids are drawn at the 80% probability level.

## supplementary crystallographic information

### Comment

Ethylenediamine has been used for approximately one century in the preparation of many metal coordination complexes, such as tris(ethylenediamine)cobalt(III) chloride (Warner, 1912). This is an important precursor to many polymers, chelating agents and pharmaceuticals, including drug design (Kotti, *et al.*, 2006). Since it is a widely used building block in the synthesis of many materials, its structure is of interest. However, it exists as a liquid at room temperature, with a melting point of 282K. We report herein the crystal structure of the dichloride salt of ethylenediamine.

Ethylenediammonium dichloride (I) crystallizes with a center of symmetry in the ethylene moiety. Fig. 1 shows the dication with one chloride counterion hydrogen bonded at each terminal nitrogen atom; however, there are three such chloride ions surrounding each N atom. The angles around both N1 and C1 are essentially tetrahedral, with the N1—C1—C1[1 - *x*,1/2 + *y*,2.5 - *z*] angle = 109.68 (18)°, and the angles around N1 range from 102 (2) to 115 (2) °.

Fig. 2 illustrates the packing of (I). Each of the chloride counterions is trigonally H bonded to three different ethylenediammonium cations with N···Cl bond distances of 3.1516 (15), 3.1931 (16) & 3.1749 (16) Å and angles N—H···Cl of 175 (2), 170 (2) & 173 (2) ° (see Table 1). Protonation occurs at both ammonium sites in the molecule (the 2nd is centrosymmetrically related); as a result, each nitrogen is also trigonally H bonded to three symmetry-related chlorides. This H bonding fixes both the chloride anions and the organic dication very rigidly in the lattice. Therefore, through symmetry, there exist six N—H···Cl bonds for each molecule, which leads to a great degree of stabilization in the structure.

### Experimental

Compound (I) was prepared by mixing 2.5 ml of ethylenediamine with 62 ml of water. Then 7.5 ml of 12 *M* HCl were added and this mixture was stirred in an ice bath at 273K until a white precipitate formed. The white precipitate was filtered and washed 3 times with methyl alcohol. The product was dissolved in water and then 12 *M* HCl was added until precipitation just began; a small quantity of water was then added to redissolve the precipitate. This mixture was allowed to evaporate slowly and large colorless needles of (I) formed, which were used directly for X-ray analyis.

### Refinement

All H atoms for (I) were found in electron density difference maps. The ammonium and methylene Hs' fractional coordinates were allowed to refine, but their isotropic thermal parameters were set at *U*_iso_(H) = 1.5*U*_eq_(N) and 1.2*U*_eq_(C).

### Crystal data

**Table:**

C_2_H_10_N_2_^2+^·2Cl^−^	*F*(000) = 140
*M**_r_* = 133.02	*D*_x_ = 1.479 Mg m^−^^3^
Monoclinic, *P*2_1_/*c*	Cu *K*α radiation, λ = 1.54178 Å
Hall symbol: -P 2ybc	Cell parameters from 1684 reflections
*a* = 4.3807 (3) Å	θ = 4.5–67.4°
*b* = 6.8569 (4) Å	µ = 8.71 mm^−^^1^
*c* = 9.9464 (5) Å	*T* = 100 K
β = 91.192 (2)°	Parallelepiped, colourless
*V* = 298.71 (3) Å^3^	0.45 × 0.30 × 0.29 mm
*Z* = 2	

### Data collection

**Table:**

Bruker SMART CCD APEXII area-detector diffractometer	521 independent reflections
Radiation source: fine-focus sealed tube	520 reflections with *I* > 2σ(*I*)
graphite	*R*_int_ = 0.022
φ and ω scans	θ_max_ = 67.8°, θ_min_ = 7.9°
Absorption correction: multi-scan (*SADABS*; Sheldrick, 2001)	*h* = −5→4
*T*_min_ = 0.085, *T*_max_ = 0.090	*k* = −8→8
1654 measured reflections	*l* = −11→11

### Refinement

**Table:**

Refinement on *F*^2^	Secondary atom site location: difference Fourier map
Least-squares matrix: full	Hydrogen site location: inferred from neighbouring sites
*R*[*F*^2^ > 2σ(*F*^2^)] = 0.028	Only H-atom coordinates refined
*wR*(*F*^2^) = 0.070	*w* = 1/[σ^2^(*F*_o_^2^) + (0.0415*P*)^2^ + 0.1865*P*] where *P* = (*F*_o_^2^ + 2*F*_c_^2^)/3
*S* = 1.15	(Δ/σ)_max_ < 0.001
521 reflections	Δρ_max_ = 0.41 e Å^−^^3^
44 parameters	Δρ_min_ = −0.31 e Å^−^^3^
0 restraints	Extinction correction: *SHELXTL* (Sheldrick, 2008), Fc^*^=kFc[1+0.001xFc^2^λ^3^/sin(2θ)]^-1/4^
Primary atom site location: structure-invariant direct methods	Extinction coefficient: 0.066 (5)

### Special details

**Table:**

Experimental. crystal mounted on cryoloop using Paratone-N
Geometry. All e.s.d.'s (except the e.s.d. in the dihedral angle between two l.s. planes) are estimated using the full covariance matrix. The cell e.s.d.'s are taken into account individually in the estimation of e.s.d.'s in distances, angles and torsion angles; correlations between e.s.d.'s in cell parameters are only used when they are defined by crystal symmetry. An approximate (isotropic) treatment of cell e.s.d.'s is used for estimating e.s.d.'s involving l.s. planes.
Refinement. Refinement of *F*^2^ against ALL reflections. The weighted *R*-factor *wR* and goodness of fit *S* are based on *F*^2^, conventional *R*-factors *R* are based on *F*, with *F* set to zero for negative *F*^2^. The threshold expression of *F*^2^ > σ(*F*^2^) is used only for calculating *R*-factors(gt) *etc*. and is not relevant to the choice of reflections for refinement. *R*-factors based on *F*^2^ are statistically about twice as large as those based on *F*, and *R*- factors based on ALL data will be even larger.

### Fractional atomic coordinates and isotropic or equivalent isotropic displacement parameters (Å^2^)

**Table:**

	*x*	*y*	*z*	*U*_iso_*/*U*_eq_	
Cl1	0.91302 (8)	0.07997 (5)	0.67014 (3)	0.0043 (3)	
C1	0.3856 (4)	0.0762 (2)	0.97532 (18)	0.0037 (4)	
H1A	0.254 (5)	0.019 (3)	0.903 (2)	0.004*	
H1B	0.269 (5)	0.131 (3)	1.047 (2)	0.004*	
N1	0.5508 (3)	0.2442 (2)	0.91610 (14)	0.0044 (4)	
H1C	0.660 (5)	0.305 (3)	0.983 (2)	0.007*	
H1D	0.444 (5)	0.328 (4)	0.885 (2)	0.007*	
H1E	0.654 (5)	0.206 (3)	0.845 (2)	0.007*	

### Atomic displacement parameters (Å^2^)

**Table:**

	*U*^11^	*U*^22^	*U*^33^	*U*^12^	*U*^13^	*U*^23^
Cl1	0.0057 (3)	0.0048 (3)	0.0023 (3)	−0.00036 (11)	−0.00138 (18)	−0.00050 (11)
C1	0.0017 (8)	0.0051 (9)	0.0041 (8)	−0.0003 (6)	−0.0013 (7)	0.0001 (5)
N1	0.0067 (7)	0.0032 (7)	0.0031 (7)	0.0013 (6)	−0.0015 (6)	0.0009 (5)

### Geometric parameters (Å, °)

**Table:**

C1—N1	1.488 (2)	N1—H1C	0.91 (2)
C1—C1^i^	1.522 (3)	N1—H1D	0.80 (3)
C1—H1A	0.99 (2)	N1—H1E	0.89 (2)
C1—H1B	0.96 (2)		
			
N1—C1—C1^i^	109.68 (18)	C1—N1—H1C	108.8 (13)
N1—C1—H1A	107.3 (12)	C1—N1—H1D	114.8 (16)
C1^i^—C1—H1A	109.4 (13)	H1C—N1—H1D	104 (2)
N1—C1—H1B	105.1 (13)	C1—N1—H1E	110.4 (15)
C1^i^—C1—H1B	112.8 (13)	H1C—N1—H1E	116 (2)
H1A—C1—H1B	112.3 (17)	H1D—N1—H1E	102 (2)

Symmetry codes: (i) −*x*+1, −*y*, −*z*+2.

### Hydrogen-bond geometry (Å, °)

**Table:**

*D*—H···*A*	*D*—H	H···*A*	*D*···*A*	*D*—H···*A*
N1—H1E···Cl1	0.89 (2)	2.27 (2)	3.1514 (15)	175 (2)
N1—H1D···Cl1^ii^	0.80 (3)	2.39 (3)	3.1770 (15)	170 (2)
N1—H1C···Cl1^iii^	0.91 (2)	2.29 (2)	3.1922 (15)	171 (2)

Symmetry codes:  (ii) −*x*+1, *y*+1/2, −*z*+3/2; (iii) *x*, −*y*+1/2, *z*+1/2.
